# Supplementary material for: Control of randomly scattered surface plasmon polaritons for multiple-input and multiple-output plasmonic switching devices
Source: Nat Commun. 2017 Mar 6;8:14636. doi: 10.1038/ncomms14636 (PMC5343438; doi:10.1038/ncomms14636)
Supplement: Supplementary Information — Supplementary Notes, Supplementary Figures and Supplementary References [file ncomms14636-s1.pdf]

Supplementary Note 1. Numerical simulation and theoretical modeling

Focusing of SPPs through a 2D disordered array of nanoholes

We performed numerical simulations and theoretical investigation on the way a 2D disordered array of nanoholes converts far-field incident waves to SPP outputs. A 100 nm-thick Au slab was numerically prepared on the top of a glass substrate, and nanoholes measuring 100 nm in diameter were patterned in an area of 5×5 μm^2 (Supplementary Figure 1a-c). The fill factor was set to be the same as experiment, which was 12 %. A monochromatic light wave with the wavelength of 620 nm was sent to the air/metal interface. The polarization of incident wave was set circular to homogenize the propagation direction of SPPs generated at the holes, and the area of the illumination was matched to the patterned area. The finite-difference time-domain (FDTD) method was used to calculate the SPPs generated at the air/metal interface until the electric fields reached the steady state. In the same way as the experiments, we constructed the transfer matrix of a disordered metal film for the far-field inputs to the SPP outputs. For each angle of illumination, the amplitude and phase maps of SPPs were recorded along the sampling line indicated as a dashed arrow in Supplementary Figure 1a. The length of sampling line was 5 μm, and the sampling interval was 100 nm. From a set of these computations, we could construct a transfer matrix $t(\eta; x, y)$, which is the complex-field amplitude of SPP at a point η for the illumination of far-field wave at a point (x, y) within the square area of the pattern.

In order to explain the effect of multiple light scattering, we considered the scanning of a rectangular beam with a width of $\Delta = 500$ nm along y-direction. At first, the illumination was placed at the edge of the patterned area that faces the sampling line (red rectangular box in Supplementary Figure 1a). This is equivalent to choosing a submatrix $t_1 = t(\eta; x, 0 \leq y \leq \Delta)$ from the full matrix t. The SPPs generated by this illumination experience little scattering on their way to the sampling line. We identified an input wave that maximizes the intensity of

SPPs at one particular position $\eta = \eta_0$ (indicated as x) from the relation, $\mathbf{E}_1 = t_1^{-1}\boldsymbol{\eta}_\mathbf{0}$. After sending this particular input wave, we recorded the complex-field map of SPPs on the metal surface (Supplementary Figure 1d) and observed that SPPs were indeed focused at the target spot. Figure S1g shows the line profile of the intensity of SPPs along the sampling line.

Next, we shifted the location of illumination by $D = 0.5$ μm away from the edge of the pattern (Supplementary Figure 1b), which is equivalent to choosing another submatrix $t_2 = t(\eta; x, D \leq y \leq D + \Delta)$. In this case, the SPPs generated at the holes illuminated by the far-field wave experience multiple scattering events on their way to the sampling line. Therefore, the attenuation of intensity is expected to occur. From the matrix, the incident wave that would maximize the SPP intensity at the target spot was identified from $\mathbf{E}_2 = t_2^{-1}\boldsymbol{\eta}_\mathbf{0}$. Similar to Supplementary Figure 1d and 1g, we observed the focusing of SPPs as shown in Supplementary Figure 1e and 1h although the intensity of the focused spot was attenuated when compared with the illumination at the edge.

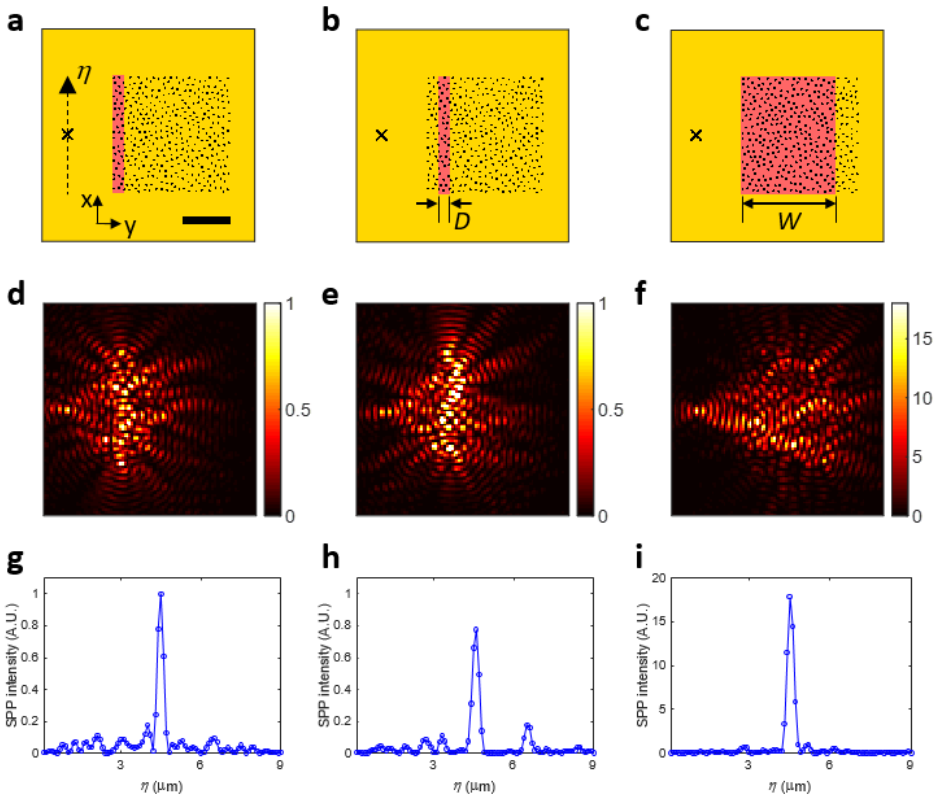

Supplementary Figure 1. Focusing of SPPs depending on the location of illumination in FDTD calculation. a-c, Sample and illumination configurations considered in the numerical calculation. Nanoholes measuring 100 nm in diameter were randomly distributed with a fill factor of 12 %. The red rectangular boxes indicate the areas where far-field waves illuminated. The dashed line with an arrow in **a** indicates SPP sampling line, and the marker x stands for a target spot where SPPs were intended to be focused. The Scale bar is 3 μm. **d-f,** Intensity maps of focused SPPs for the cases of **a-c**, respectively. The same arbitrary unit was applied to the color bars. **g-i,** Line profiles along the sampling lines in **d-f**, respectively.

Theoretical investigation of the increase of channel number

In accordance with the experiments, we considered the increase of the width of illumination and quantified the signal to noise ratio (SNR) of the SPP focusing with respect to W. We gradually increased W (Supplementary Figure 1c) by choosing the submatrix $t_3 = t(\eta; x, 0 \leq y \leq W)$, and obtained the complex-field map of SPPs focused to the target spot (Supplementary Figure 1f). As shown in the intensity profile along the sampling line

(Supplementary Figure 1i), the intensity has increased by about 13 times when the width of illumination was increased to W = 8Δ. The intensity of the target spot has steadily increased with the increase of W (Supplementary Figure 2c) even though the intensity of focused spot has steadily decreased with respect to D (Supplementary Figure 2a). This is because the SPPs originating from slabs with different D have constructively interfered at the target spot. The red dots in Supplementary Figure 2c were the intensities at the target spot calculated by the coherent addition of the waves from the intensity response as a function of D in Supplementary Figure 2a, and these are in good agreements with those measured as a function of W (blue dots in Supplementary Figure 2c).

Next, we analyzed the SNR of SPP focusing, which is an important parameter that determines the robustness of the communications and also effectively the information transfer capacity. As shown in Supplementary Figure 2d, the SNR increases steadily with the increase of W and reached to about 3 times of that by an individual segment of width Δ. This has a significant gain in the information transfer capacity. The SPPs are well randomized at every 0.8 μm in D. The red dots in Supplementary Figure 2d is the SNR predicted from the assumption that the SPPs are completely decorrelated when D is increased by 0.8 μm. The blue dots are the measured SNR. The discrepancy arises from the fact that the SPPs are not totally randomized, and there exist residual correlation of around 0.2 as shown in Supplementary Figure 2b. These analyses support the role of multiple light scattering in enhancing the SNR.

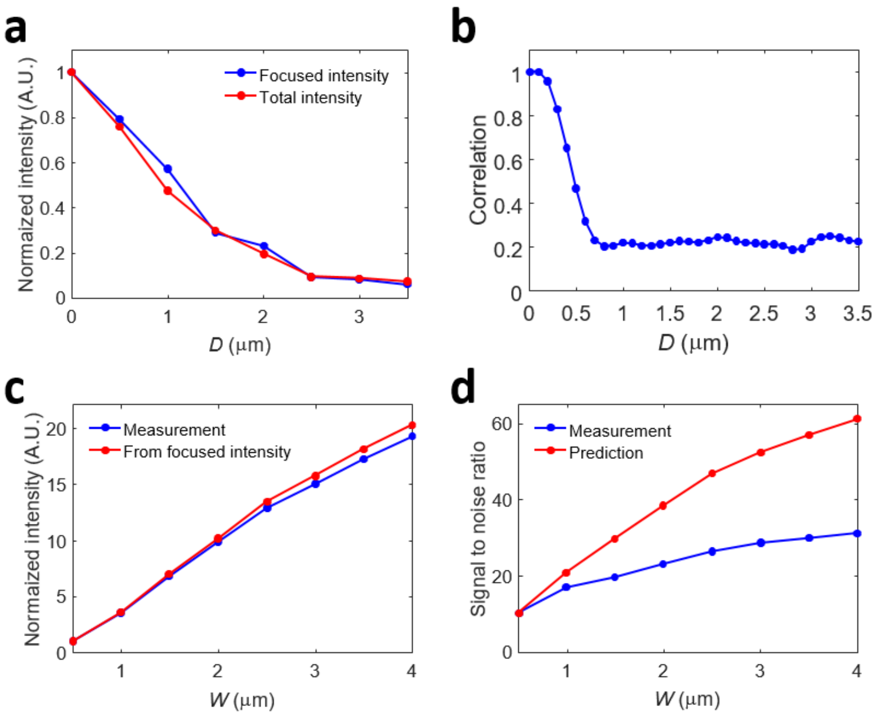

Supplementary Figure 2. Intensity at a target spot and signal to noise ratio with the increase of illumination beam width. a, Intensity on a focused spot depending on D. The blue dots were intensities at the target spots measured in Supplementary Figure 1d-e, and the red dots the total intensity of SPPs measured along the sampling line. Both were normalized by the values at D = 0. **b,** Normalized cross-correlation of SPPs originating from different D with respect to those at D = 0. **c,** The intensity at a focused spot depending on W. The blue dots were intensities measured at target spot in Supplementary Figure 1f, and the red dots were calculated from **a**. **d,** Signal to noise ratio with respect to W. The blue dots were from the line profile in Supplementary Figure 1i, and the red dots were the prediction from **a** assuming that the SPPs from different D are fully uncorrelated.

Supplementary Note 2. Decorrelation of SPPs due to multiple light scattering

Calculation of the cross-correlation of SPPs in Fig. 4d

Due to the limitation of space in the figure caption, only a brief explanation was given for Fig. 4d. The data points in the figure were the magnitude of the cross-correlation of the SPPs originating from the segment of nanoholes at $0 \leq y \leq 100$ nm with those from $D \leq y \leq D +$ 100 nm. The mathematical procedure is given in detail in the following. The SPP field at the

sampling line $\boldsymbol{\eta}_{\mathbf{1}}(\eta)$ can be calculated for the illumination of far field wave $\mathbf{E}_{\boldsymbol{n}}$ at the slab $0 \le y \le 100$ nm from the measured transmission matrix t:

$$\boldsymbol{\eta}_{\mathbf{1}}(\eta) = t(\eta; x, 0 \le y \le 100\ \text{nm}) \times \mathbf{E}_{\boldsymbol{n}}(x, 0 \le y \le 100\ \text{nm}). \quad (1)$$

Here $\mathbf{E}_{\boldsymbol{n}}$ can be arbitrary, but we chose it to be normally incident plane wave such that the amplitude is the same for all the elements within the segment defined by $(0 \le x \le L, 0 \le y \le 100\ \text{nm})$. Likewise, the SPP field $\boldsymbol{\eta}_{\mathbf{2}}(\eta)$ generated by the far field illumination $\mathbf{E}_{\boldsymbol{n}}$ at the slab $D \le y \le D + 100$ nm can be calculated by the following calculation:

$$\boldsymbol{\eta}_{\mathbf{2}}(\eta) = t(\eta; x, D \le y \le D + 100\ \text{nm}) \times \mathbf{E}_{\boldsymbol{n}}(x, D \le y \le D + 100\ \text{nm}). \quad (2)$$

Then, the normalized cross-correlation of the two SPP fields was calculated by the following equation:

$$C(D) = \frac{\langle \boldsymbol{\eta}_1^* \cdot \boldsymbol{\eta}_2 \rangle}{\|\boldsymbol{\eta}_1\| \cdot \|\boldsymbol{\eta}_2\|}. \quad (3)$$

Incorporation of residual correlation in estimating α

The enhancement factor of channel number that our theoretical model,

$$\alpha = \frac{N_{\text{eff}}^{2\text{D}}}{N^{1\text{D}}} = \frac{\left|\sum_{j=0}^{m-1} \sqrt{T(jl_{\text{c}})}\right|^2}{\sum_{j=0}^{m-1} T(jl_{\text{c}})}, \quad (4)$$

predicts for the given experimental parameters ($L = 10\ \mu\text{m}$, $l_{\text{c}} = 1\ \mu\text{m}$, $l_{\text{a}} \approx 2.4\ \mu\text{m}$, and $l_{\text{t}} \approx 7.2\ \mu\text{m}$) was $\alpha_{\text{th}} \approx 9$. On the other hand, the experimentally measured enhancement factor presented in Fig. 4c was $\alpha_{\text{exp}} \approx 6$. This discrepancy was mainly due to the residual correlation of SPPs originating from different segments in the disordered array of nanoholes. As shown in Fig. 4d, this residual correlation was measured to be about 0.2. We accounted for this correlation in estimating α, and the modified model predicts the enhancement factor of around 6 (red dots in Fig. 1e), a good agreement with the experimental results.

Here we explain the detailed procedure to include its effect on α. A transfer matrix $t_{\text{num}}(\eta;\ x, y)$ was generated numerically using random matrix theory in which the columns

of the matrix are completely orthogonal with respect to each other. We then applied the attenuation of the SPPs depending on y following the transmittance function $T(y)$. The same vector was added to each column in such a way that approximately 20 % of residual cross-correlation existed among columns of different y. Finally, we used this matrix to calculate the enhancement factor, which is shown as red dots in Fig. 1e.

Supplementary Note 3. The potential layout of the optoelectronic MIMO network using disordered array of nanoholes

The general layout of MIMO network used in wireless communications is described below.

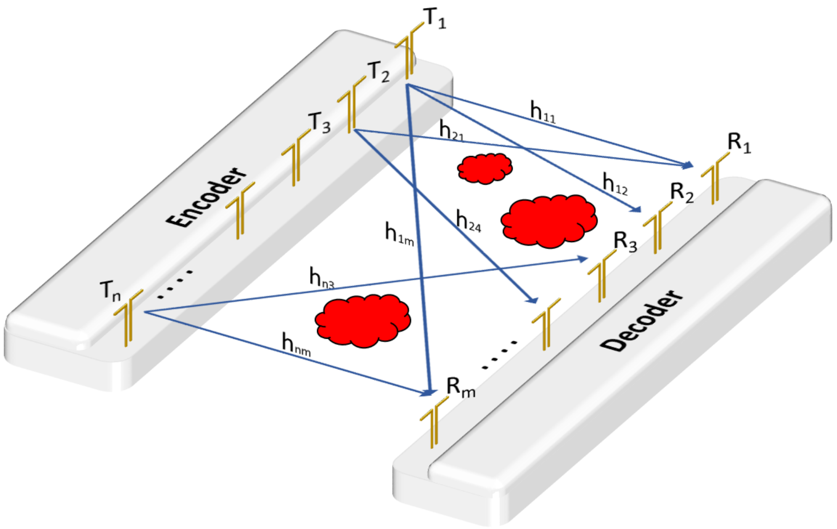

Supplementary Figure 3. Typical layout of multiple-input multiple-output network. $T_1, T_2, \cdots$ are the transmitters and $R_1, R_2, \cdots$ are the receivers. The h_{ij} stands for the amplitude transmittance from i^{th} transmitter to the jth receiver.

Typically, a number of transmitters $(T_1, T_2, \cdots, T_n)$ and receivers $(R_1, R_2, \cdots, R_m)$ are spatially multiplexed to enhance channel capacity. Spatial multiplexing is made possible due to the multiple scattering of waves emanating from transmitters on their way to receivers by means of random reflections from walls and clouds. In our case, the disordered array of nanoholes

plays a similar role. In order to decode the multiplexed signals, the inversion of the transfer matrix H, whose elements h_{ij} are the amplitude transmittance from i^{th} transmitter to j^{th} receiver, is used. In the real-world wireless communications, null data is sent from transmitters to receivers to measure H prior to the transfer of information. This is exactly the same as our measurements of transfer matrix.

For a comparison, we suggest a possible optoelectronic network in Supplementary Figure 4 that our device of the disordered array of nanoholes can be integrated into. The disordered array of nanoholes may sit in the middle, and multiple electronic devices (output devices 0-6 in this example) can be located around. The multiple input device, which is the spatial light modulator in our study, controls far-field channels and therefore corresponds to the transmitters in MIMO network. The electronic devices located around the disordered array of nanoholes are equivalent to the receivers. Therefore, the number of spots to be used for the control will depend on the number of electronic devices to be connected to. And the maximum number of devices that can be connected to will be determined by the channel capacity, which is 40 in our case.

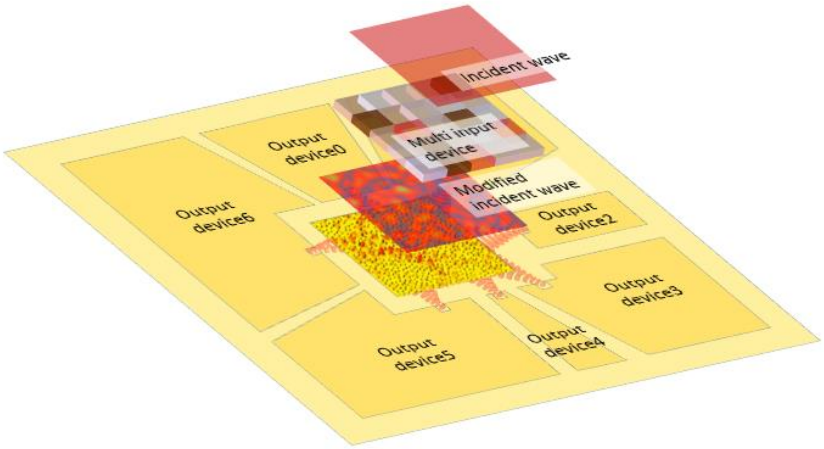

Supplementary Figure 4. The exemplary layout of the optoelectronic MIMO network by using the disordered array of nanoholes as a switching device.

In many aspects, our device has similar layout with the conventional MIMO network, but there exists important difference in the types of signals. Our device mediates far-field waves

propagating in 3D to SPPs propagating in 2D, while the ordinary MIMO network concerns far-field waves for both transmitters and receivers.

Supplementary Note 4. Scattering properties of 2D disordered array of nanoholes

Scattering mean free path of SPPs propagating through disordered array of nanoholes

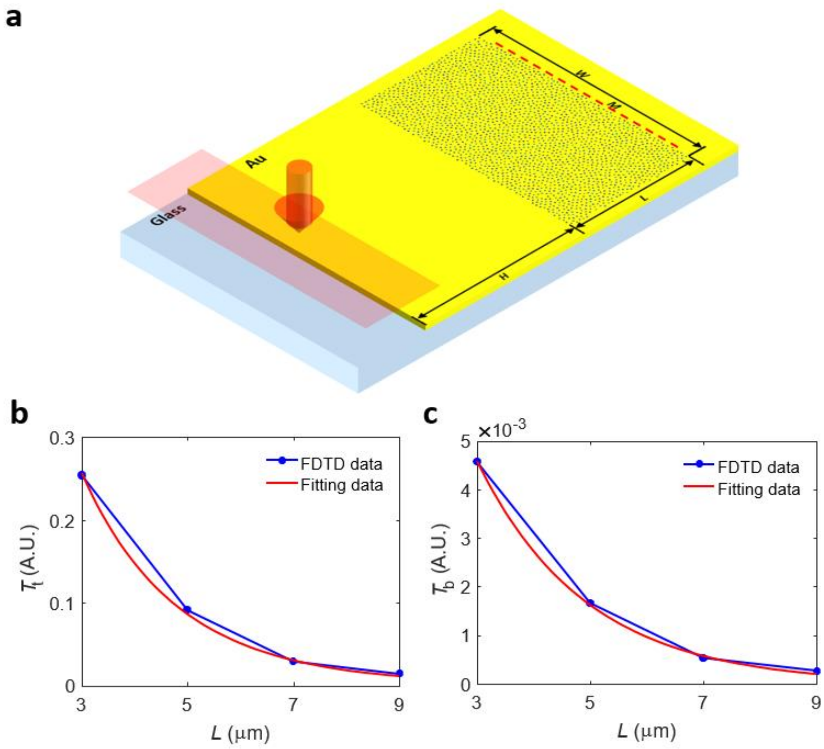

Supplementary Figure 5. Determination of the scattering mean free path and absorption length. a, Schematic diagram for the geometry of a sample and beam illumination in FDTD simulation. The fill factor of nanoholes is 12 %. Far-field wave illuminated the metal edge, where SPPs with planar wavefront parallel to the edge were generated and propagated toward the disordered array of nanoholes. H = 12 μm is the distance from the edge to the nanohole pattern. L and W = 16 μm are respectively the thickness and width of the disordered array of nanoholes. M is a detection line for measuring the SPP transmission. **b,** Total transmittance of SPPs from the FDTD simulation depending on the thickness L (blue dots). The red curve shows data fitting to theoretical function $T(L)$. **c,** Thickness-dependent intensity of the ballistic component of the transmitted SPPs that have the same wavefront as that of the

incident SPP to the disordered pattern. Red curve stands for exponential curve fitting with the decay length l' = 1.7 μm.

In general, three length parameters are used for characterizing the propagation of waves through a disordered medium. These are the absorption length l_{a}, scattering mean free path l_{s}, and transport mean free path $l_{\mathrm{t}} = l_{\mathrm{s}}/(1-g)$, where g is an anisotropy factor[1]. In our case of the interaction between SPPs and the 2D disordered array of nanoholes, the absorption length l_{a} are determined by the metallic loss and scattering loss. The metallic loss is caused by the finite conductivity of metal, and the scattering loss is caused by the out-of-plane scattering to the far-field wave. The l_{s} and l_{t} are determined by the alteration of SPPs in their directions due to the scattering at the nanoholes.

In order to determine these three parameters, we measured both the total transmittance and the transmittance of ballistic components of SPPs propagating through the 2D disordered array of nanoholes for various thicknesses L of the pattern. Supplementary Figure 5a is the schematic diagram of the numerically prepared sample. We put 100 nm-thick Au film on the glass substrate and then made disordered array of nanoholes. The fill factor and the diameter of the holes were set to be the same as those used in experiment. We varied the thickness L of the pattern from 3 μm to 9 μm with an interval of 2 μm. A light source illuminates the metal edge, where SPPs were generated, propagated through the disordered pattern and were detected at the red dashed line M. The generated SPPs have wavefront parallel to the edge so that they make a normal incidence to the disordered pattern.

The total transmission T was measured depending on the thickness L (blue dots in Supplementary Figure 5b). Theoretically, the total transmittance is related to l_{a} and l_{t} by the following relation, $T(L) \propto \frac{l_{\mathrm{t}}}{L+c} exp(-L/l_{\mathrm{a}})$ [2], where c is an extrapolation lengths. The red curve in Supplementary Figure 5b is the fitting of measured data by this total transmittance function. In this fitting, l_{a} was determined to be 2.4 μm. We then measured the transmittance of the ballistic component, $\mathrm{T_b}$ (blue dots in Supplementary Figure 5c). This data was fitted by

the theoretical function, $T_{\mathrm{b}}(L) \propto exp(-L/l')$ [1] (red curve), where total attenuation length l' defined by $1/l' = (1/l_{\mathrm{a}} + 1/l_{\mathrm{s}})$ was determined as 1.74 μm. From the previously determined l_{a}, we could determine the scattering mean free path, l_{s} = 6.3 μm. Since the g was calculated to be 0.12 from the scattering pattern of an individual hole, we could determine the transport mean free path l_{t} = 7.2 μm.

Determination of the characteristic length l_{c}

As mentioned in the main text, multiple light scattering occurring within the disordered array of nanoholes gives rise to two major effects — the scattering loss and the decorrelation of SPPs. We measured the characteristic length l_{c} that describes the length scale of these two effects. Let us first take a look at the scattering loss. Supplementary Figure 2a shows the intensity of the focused spot as a function of D. The intensity was decreased almost exponentially due to the multiple light scattering, and the decay constant of about 1.4 μm. The measured decay constant in Supplementary Figure 2a was similar to $T(L)$, but somewhat due to the finite size of the patterned area. SPPs from large D leaks out of the pattern, and the intensity decay appears to be more pronounced than the case of the infinite slab where scattering mean free path is estimated.

Let us then look at the decorrelation effect. The SPPs originating from different segments are largely uncorrelated due to multiple light scattering. We measured the length scale of this decorrelation (Supplementary Figure 2b). The SPPs, $E(\eta; x, D \leq y \leq D + \Delta)$, were measured along the sampling line for the normally incident plane wave. And the normalized cross-correlation of SPPs for $D \neq 0$ with respect to $D = 0$ was calculated as a function of D. As shown in Supplementary Figure 2b, the correlation decreased to the baseline at a distance of about 0.8 μm. Therefore, the SPPs are well randomized at every 0.7 μm in D. Therefore, we defined this length as the characteristic length l_{c} governing the interaction of SPPs with

disordered nanoholes. With additional simulations, we confirmed that l_c is increased for the increased numerical aperture of the illumination.

Effects of absorption and scattering losses to the enhancement factor

The enhancement factor is almost equal to m for small m because the attenuation of $T(y)$ is relatively weak for the SPPs generated at the segments near the sampling line. However, the attenuation in $T(y)$ is not negligible for large m. Therefore, the enhancement factor becomes smaller than m as the size of the device is increased.

The attenuation of $T(y)$ is determined by l_a and l_t following the relation, $T(y) = \left(l_\mathrm{t}/(y+c)\right)exp(-y/l_\mathrm{a})$. Now the question is to what degree the l_t contributes to the enhancement factor. If we consider only l_a, the transmittance is given simply by $T_1(y) = exp(-y/l_\mathrm{a})$. As shown in Supplementary Figure 6a, $T(y)$ is smaller and decays faster than $T_1(y)$. As a consequence, the enhancement factor is smaller when l_t is included (Supplementary Figure 6b). However, the simple case of $T_1(y)$ agrees quite well up to $m = 4$ with the case of $T(y)$. Therefore, the simple inclusion of l_a should be good enough for small m. Indeed, the effect of l_t is pronounced mainly at large m.

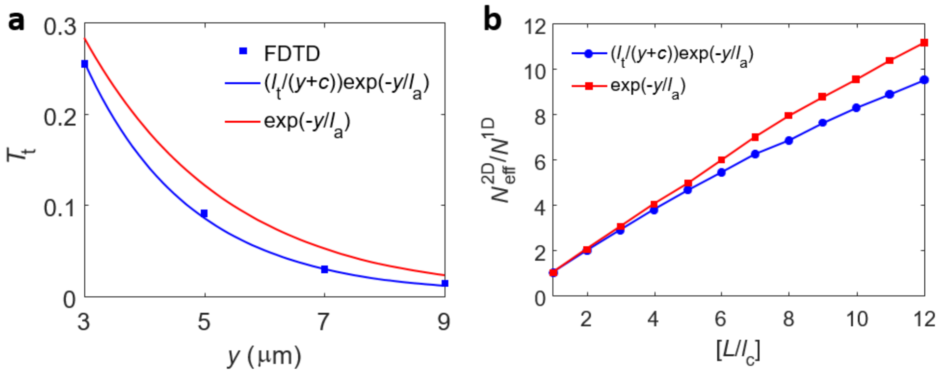

Supplementary Figure 6. The effects of absorption and scattering losses to the channel enhancement factor. a, Transmittance of SPPs for a distance y. Blue dots: FDTD simulation, blue curve: $T(y)$, red curve: $T_1(y)$. **b,** Theoretical estimation of the channel enhancement

factor as a function of the size of the device for the cases of $T(y)$ (blue dots) and $T_1(y)$ (red dots).

Supplementary Note 5. Additional experimental details

The effects of fill factor to the enhancement factor

We investigated the effect of the fill factor by performing experiments for the samples with various fill factors. Supplementary Figure 7 shows the enhancement factor of samples whose fill factors were ranging from 3 to 15 %. The enhancement factor was increased up to 12 %, and then decreased at 15 %.

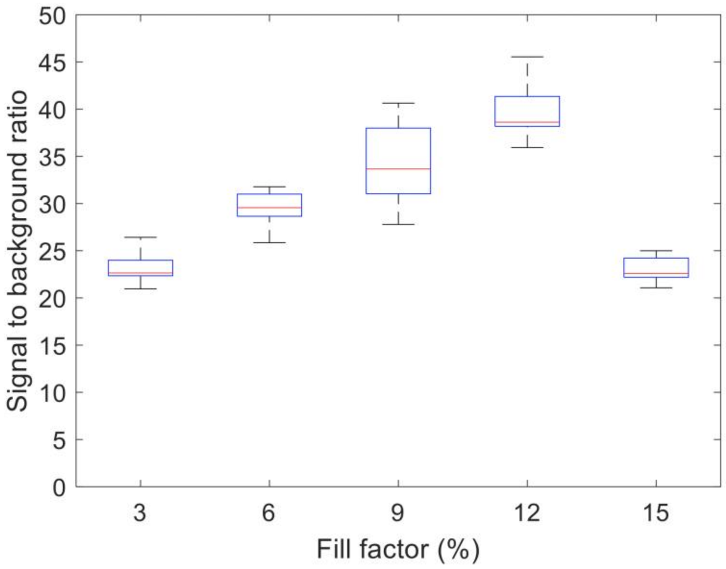

Supplementary Figure 7. Signal to background ratio, or the enhancement factor, for various fill factors. The size of all the samples was $L = 10\ \mu\text{m}$. For each device, enhancement factors were measured at different target spots along the sampling line, and the statistical distribution were displayed by the standard box plot.

According to our theoretical model, the channel number can be described by the following relation: $N_{\text{eff}}^{2\text{D}} = \alpha N^{1\text{D}} = \frac{\left|\sum_{j=0}^{m-1}\sqrt{T(jl_c)}\right|^2}{\sum_{j=0}^{m-1}T(jl_c)}N^{1\text{D}}$. Here the fill factor affects to the enhancement factor $N^{1\text{D}}$ per segment of $L \times l_c$ and the transmittance of SPPs $T(y)$ from holes located at y

to the sampling line. In order to reach the theoretical $N^{1\mathrm{D}}$, the fill factor should be larger than approximately 3 % at the very least for the given experimental condition, and in fact the higher the better. Otherwise, there are too a small number of nanoholes that can convert far-field channels to those of SPPs. On the other hand, the increase in the fill factor results in the steep decrease of $T(y)$ due to the scattering loss, thereby reducing $N_{\mathrm{eff}}^{2\mathrm{D}}$.

But there exists additional important effect that is determined by the fill factor. The disordered array of nanoholes makes the contribution of SPPs from different segments independent. This was made clear in the cross-correlation plot shown in Fig. 4d. Even with the use of the disordered array, however, there exists residual correlation which reduces the effective number of converted channels. At higher fill factor, the residual cross-correlation among segments is smaller. As a result, the SPPs from different segments are more independent such that the enhancement factor approaches to the theoretical expectation. Accounting for all these three effects that the fill factor gives rise to, there exists a certain fill factor for the optimum channel conversion efficiency, which was about 9-12 % for our experimental condition.

The effect of the residual far-field waves

In order to assess the influence of the residual far-field waves, we performed a quantitative analysis on the relative intensities of the far-field waves and SPPs. The typical intensity map taken at the back aperture of the objective lens (the inset image in Fig. 2) shows that there were far-field waves as well as SPPs in the leakage radiation microscope. As explained in the main text, we placed a beam block to minimize the intensity of the far-field waves at the camera. Supplementary Figure 8a shows the typical field map recorded at the camera. The angular intensity distribution of this detected wave is shown in Supplementary Figure 8b in which the bright ring that corresponds to k_{SPP} was the dominant signal. From this data, we analyzed the relative intensity between SPPs and far-field waves, and observed that the total

residual far-field waves were about 10 % of SPPs. This suggests that the far-field waves contributed little to the control of SPPs.

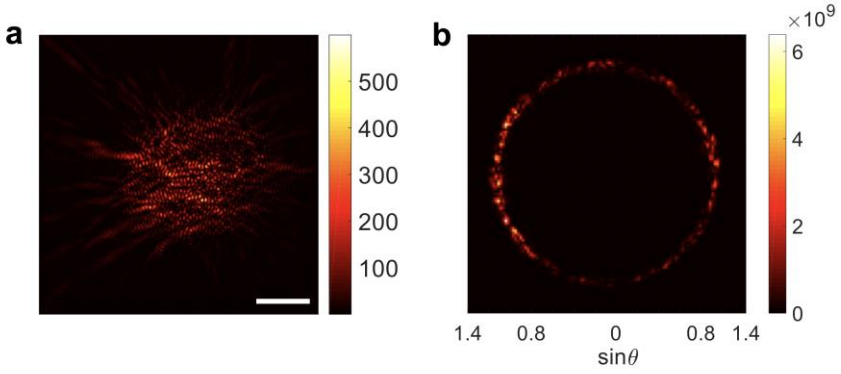

Supplementary Figure 8. Comparison between SPPs and far-field waves. a, Typical intensity map measured by the interferometric leakage radiation microscope. Color bar, intensity in arbitrary unit. Scale bar, 5 μm. **b,** Fourier transform of the complex field map in **a**, which corresponds to the angular distribution of detected field. θ indicates azimuthal angle of scattered wave. Bright ring corresponds to k_{SPP}. Color bars, intensity in arbitrary unit.

We also analyzed the contribution of the surface roughness by performing additional experiments for the samples with no nanoholes. Although there existed SPPs from the surface, its intensity was much smaller than those from nanoholes. Supplementary Figure 9 shows the optimized intensity at the target as a function of the width of illumination for the sample with no nanoholes (square dots) in comparison with the sample of 12 % fill factor (circular dots). According to these measurements, the contribution of the surface roughness was measured to be less than 1 % at the full width of illumination. Therefore, we could confirm that SPPs from the nanoholes were the main source of signals in our experiment.

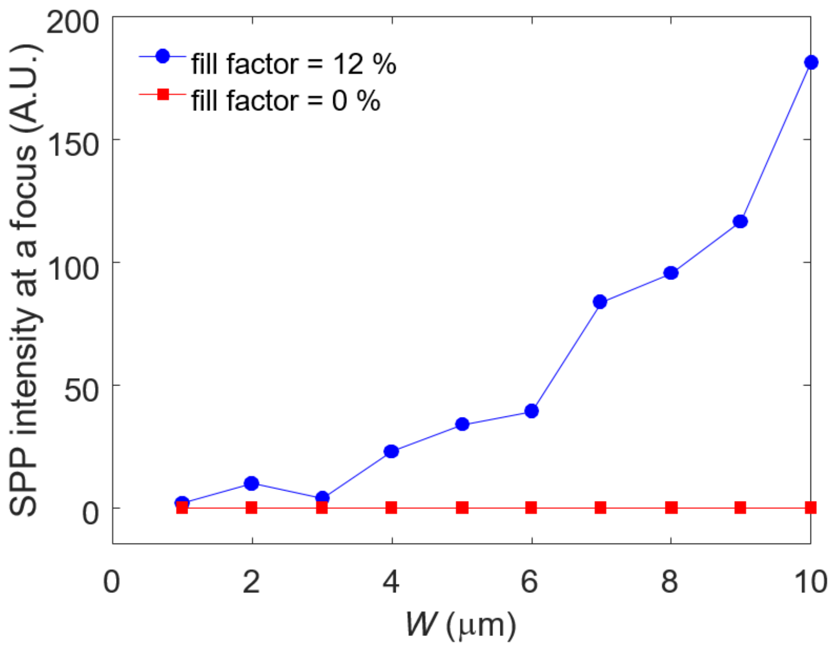

Supplementary Figure 9. Control experiment using the sample with no nanoholes. The intensity of the optimized spot was measured as a function of the width of illumination (square dots). As a comparison, the same data is shown for the sample with the 12 % fill factor of nanoholes (circular dots). The two curves were displayed at the same scale for the direct comparison of their intensities.

Construction of a transfer matrix from experimentally measured SPP maps

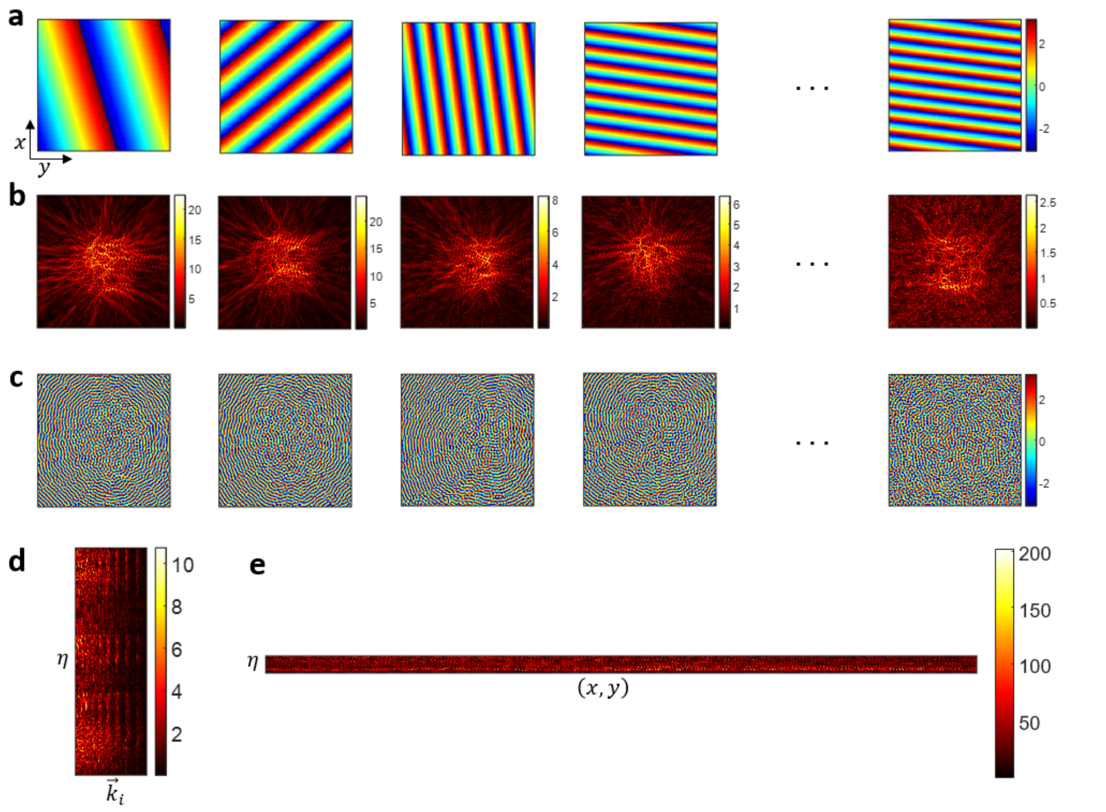

Supplementary Figure 10. Experimental construction of a transfer matrix. a, Phase ramps of incident wave written on the SLM. Each image corresponds to each transverse wavevector $\vec{k}_i$ of the illumination. Color bar, phase in radians. **b,** Amplitude maps of SPPs measured at the camera. Scale bar, 5 μm. Color bar, amplitude in arbitrary unit. **c,** Phase maps of SPPs measured at the camera. Color bar, phase in radians. **d,** Transfer matrix, $t(\eta;\vec{k}_i)$ reconstructed from **a-c**. The columns are $\vec{k}_i$, and rows the complex amplitude of SPPs along the sampling line. **e,** Transfer matrix $t(\eta;x,y)$ after converting the basis from $\vec{k}_i$ to (x,y) using the relation between $\vec{k}_i$ and (x,y) shown in **a**.

Focusing of SPPs using 1D array of nanoholes

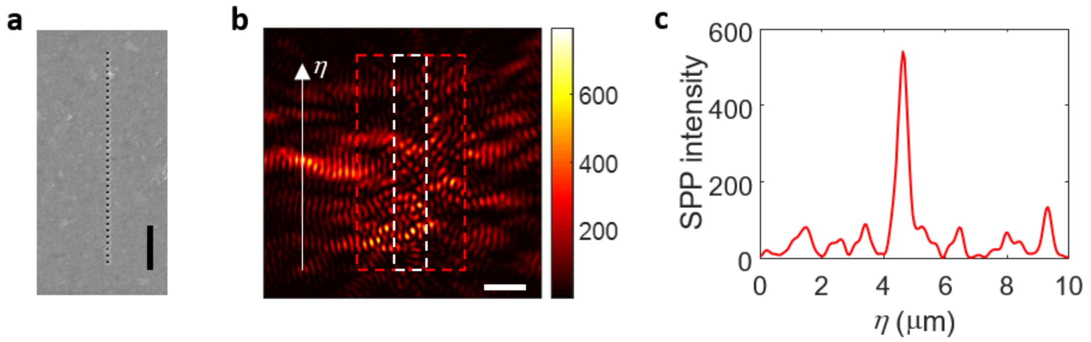

Supplementary Figure 11. Focusing of SPPs using a 1D periodic array of nanoholes. a, Nanoholes patterned on an Au film. The interval between the neighboring holes is 300 nm. Scale bar, 2 μm. **b,** Experimentally implemented focusing of SPPs to a target spot. White rectangular box indicates where nanoholes are located, and red box is where the far-field wave illuminated. In the same way as the focusing experiments for the 2D disordered array of nanoholes, the transfer matrix of a 1D periodic nanoholes was first measured, and then the pattern that maximizes the intensity of SPP to the target spot was identified and written on the SLM. Scale bar, 2 μm. **c,** Line profile along the sampling line. The measured SNR was 7.6.

Additional experiments for $L = 5$ μm samples

We also performed additional experiments for $L = 5$ μm sample at the same 12 % fill factor as $L = 10$ μm sample (Supplementary Figure 12). The slope was smaller at $L = 5$ μm than $L = 10$ μm because the enhancement factor $N^{1\mathrm{D}}$ per segment of $L \times l_{\mathrm{c}}$ is smaller. In addition, there were only 5 effective segments (the effective number of segment is given by $m = [L/l_{\mathrm{c}}]$) in $L = 5$ μm sample while there were 10 in $L = 10$ μm sample. Due to these combined effects, the enhancement factor for $L = 5$ μm sample was smaller than that of $L = 10$ μm. But the behavior of the increase of channel conversion efficiency was similar.

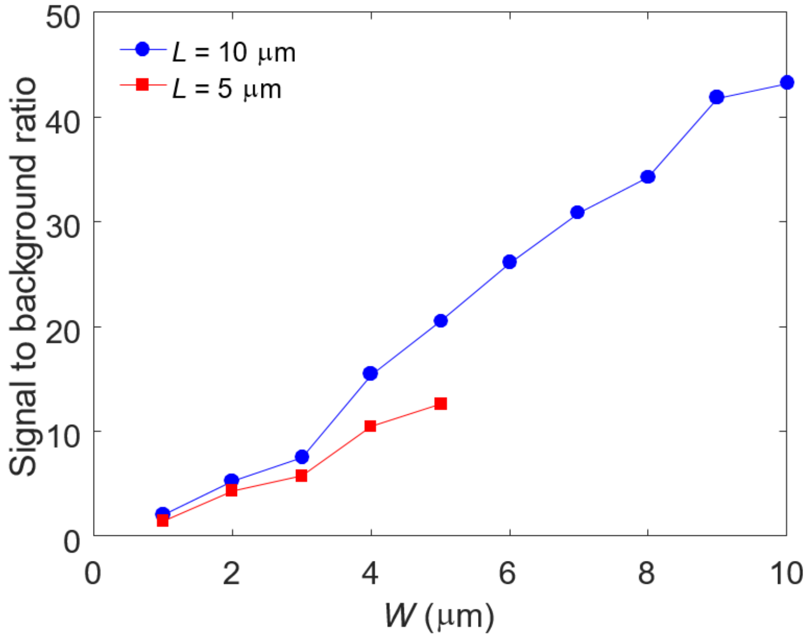

Supplementary Figure 12. Enhancement factor for $L = 5$ μm and $L = 10$ μm samples as a function of the width of illumination. Both samples have the same 12 % fill factor.

Supplementary Note 6. Numerical study comparing the use of a silver layer with that of a gold layer

The loss of SPPs is one of the governing factors for the channel enhancement factor. Therefore, the use of silver layer as a host medium and illumination of light wave with longer wavelength will be beneficial to the channel conversion efficiency. To investigate the effect of SPP loss, we performed additional numerical simulation for a sample made of a silver layer using the wavelength of light source $\lambda = 800$ nm. For a direct comparison with the gold sample, the fill factor of the nanoholes was set to 12 %. As shown in Supplementary Figure 13, however, the enhancement factor of the silver sample (black dots) was smaller than that of the gold sample (blue dots). It turned out that the use of longer wavelength did more harm than good. The correlation length l_c measured by the width of the cross-correlation curve was made longer because the effective diffraction limit spot along the y-direction was larger. Therefore, the effective number of slabs $m = [L/l_c]$ was smaller for the silver sample at

$\lambda = 800$ nm. Another disadvantage of using longer wavelength is that the channel number N^{1D} per segment of $L \times l_c$ was smaller as the effective number of diffraction limit spots was reduced along the x-direction in the slab. This can be seen at $W = 0.5$ μm in Supplementary Figure 13 in which the signal to noise ratio for Au at $\lambda = 620$ nm was larger than that for Ag at $\lambda = 800$ nm.

We therefore performed additional simulation for the same silver sample, but at the same wavelength $\lambda = 620$ nm as gold sample. As shown by the red dots in Supplementary Figure 13, the enhancement factor of the silver sample was now larger than that of the gold sample. When W was increased, the increase in the enhancement factor was rather linear in the silver sample while the slope of enhancement factor was decreased in gold sample. This was mainly due to the reduced loss of SPPs in the silver sample. These additional studies suggest that the use of low loss sample is preferable for the maximal channel transfer.

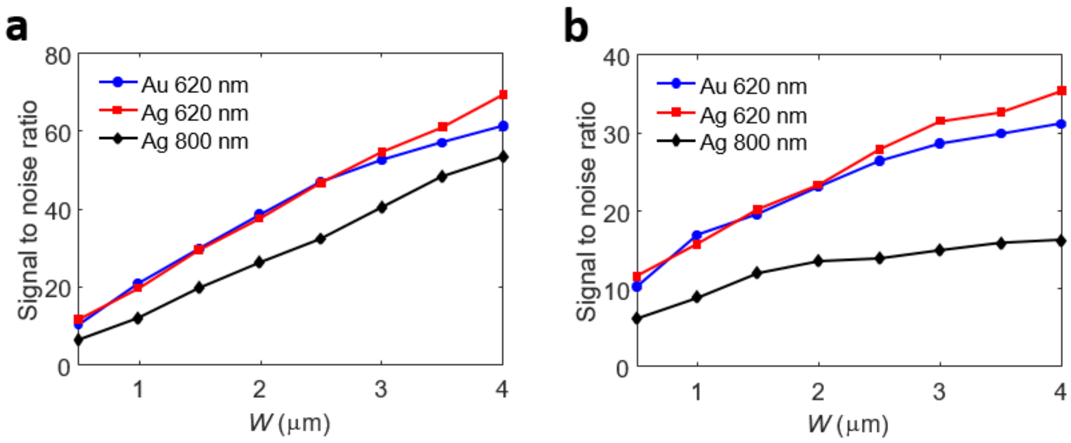

Supplementary Figure 13. Numerical study comparing silver layer with gold layer. a, Signal to noise ratio with respect to W predicted from the calculated $T(y)$. Black and red dots are from silver samples at the wavelength of 800 nm and 620 nm, respectively. Blue dots are from the gold sample at the wavelength of 620 nm. **b,** Signal to noise ratio measured by the numerical focusing. Black, red and blue dots indicate the same conditions as those specified in **a**. The discrepancy between the measurements and predictions was due to the residual correlation discussed in Supplementary Note 1.

Supplementary References

1. Andrews DL. *Photonics. Volume 4, Biomedical photonics, spectroscopy, and microscopy*.

2. Cai W, Xu M, Lax M, Alfano RR. Diffusion coefficient depends on time, not on absorption. *Opt Lett* **27**, 731-733 (2002).
